# Does Developing Interpregnancy Hypertension Affect the Recurrence Risk of Preeclampsia? A Population-Based Cohort Study

**DOI:** 10.1093/ajh/hpae034

**Published:** 2024-03-19

**Authors:** Inger Björk Ragnarsdóttir, Tansim Akhter, Katja Junus, Linda Lindström, Susanne Lager, Anna-Karin Wikström

**Affiliations:** Department of Women’s and Children’s Health, Uppsala University, Uppsala, Sweden; Department of Women’s and Children’s Health, Uppsala University, Uppsala, Sweden; Department of Women’s and Children’s Health, Uppsala University, Uppsala, Sweden; Department of Women’s and Children’s Health, Uppsala University, Uppsala, Sweden; Department of Women’s and Children’s Health, Uppsala University, Uppsala, Sweden; Department of Women’s and Children’s Health, Uppsala University, Uppsala, Sweden

**Keywords:** blood pressure, cardiovascular disease, chronic hypertension, hypertension, preeclampsia, pregnancy

## Abstract

**BACKGROUND:**

Preeclampsia in a first pregnancy is a strong risk factor for preeclampsia in a second pregnancy. Whether chronic hypertension developed after a first pregnancy (interpregnancy hypertension) affects the recurrence risk of preeclampsia is unknown.

**METHODS:**

This is a population-based cohort study of 391,645 women with their first and second singleton births between 2006 and 2017. Exposure groups were women with preeclampsia in their first pregnancy, interpregnancy hypertension, or both risk factors. Women with neither risk factor were used as a reference group. We calculated the adjusted relative risk (aRR) with 95% confidence intervals (CIs) for overall preeclampsia in the second pregnancy as well as preterm (<37 gestational weeks) and term (≥37 gestational weeks) subgroups of the disease.

**RESULTS:**

Women with preeclampsia in their first pregnancy who did or did not develop interpregnancy hypertension had rates of preeclampsia in their second pregnancy of 21.5% and 13.6%, respectively. In the same population, the corresponding rates of preterm preeclampsia were 5.5% and 2.6%, respectively. After adjusting for maternal factors, women with preeclampsia in their first pregnancy who developed interpregnancy hypertension and those who did not have almost the same risk of overall preeclampsia in their second pregnancy (aRRs with 95% CIs: 14.51; 11.77–17.89 and 12.83; 12.09–13.62, respectively). However, preeclampsia in the first pregnancy and interpregnancy hypertension had a synergistic interaction on the outcome of preterm preeclampsia (aRR with 95% CI 26.66; 17.44–40.80).

**CONCLUSIONS:**

Women with previous preeclampsia who developed interpregnancy hypertension had a very high rate of preterm preeclampsia in a second pregnancy, and the two risk factors had a synergistic interaction.

## Graphical Abstract

**Figure:**
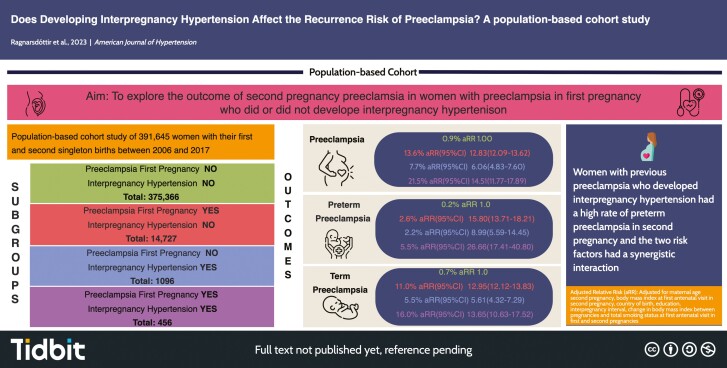
This graphical abstract is also available at Tidbit: https://tidbitapp.io/tidbits/does-developing-interpregnancy-hypertension-affect-the-recurrence-risk-of-preeclampsia-a-population-based-cohort-study-e7ae61bd-d8c5-4a8b-9fab-faaf023de733.

Preeclampsia complicates 3%–5% of pregnancies worldwide and is among the leading causes of maternal and neonatal morbidity, accounting for over 50,000 maternal deaths annually. Preeclampsia is generally defined as new-onset hypertension after 20 gestational weeks, accompanied by signs of end-organ damage.^1^ The pathophysiology of preeclampsia is a complex interaction of genetic, epigenetic, lifestyle, and environmental factors.^2^ There are several well-known risk factors for preeclampsia, such as nulliparity, high body mass index (BMI), high maternal age, and certain chronic diseases, for example, chronic hypertension.^3,4^ Women with previous preeclampsia have an increased risk of recurrence in subsequent pregnancies and are predisposed to chronic hypertension and other cardiovascular diseases later in life.^5–7^ The association between increased risk of long-term chronic hypertension and cardiovascular disease is stronger for preterm (<37 gestational weeks) than term (≥37 gestational weeks) preeclampsia.^8,9^

In early pregnancy, women are risk assessed for adverse pregnancy outcomes, such as preeclampsia. Women identified as high risk for preeclampsia may be offered aspirin to prevent preeclampsia and increased surveillance during pregnancy for early detection of preeclampsia.^10^ Most international guidelines recognize previous preeclampsia and chronic hypertension as major risk factors for preeclampsia.^11^ Several studies have explored the relationship between various hypertensive disorders of pregnancy in consecutive pregnancies.^12–14^ However, a limited number of studies have focused on the risk of superimposed preeclampsia, that is, preeclampsia occurring in women with chronic hypertension, in women with previous preeclampsia.^8,15,16^ Not much is known about the interaction between previous preeclampsia and interpregnancy development of chronic hypertension on the risk of overall preeclampsia, as well as preterm and term subtypes. The recurrence risk of preeclampsia is around 14%–21%.^17,18^ The prevalence of chronic hypertension in pregnancy has been increasing in recent years, and up to 30%–50% of women with previous preeclampsia may develop chronic hypertension within a decade.^19^ Thus, an increasing number of women with preeclampsia in their first pregnancy may develop chronic hypertension before their second pregnancy. Investigating the interaction between preeclampsia in a first pregnancy and interpregnancy hypertension on recurrence risk in a second pregnancy is therefore of importance.

Further, considering that preterm and term preeclampsia differ in pathophysiology, the two risk factors, previous preeclampsia, and interpregnancy hypertension, might interact differently depending on the subtype of preeclampsia.^20,21^

We have conducted a nationwide register-based cohort study to explore the risk of preeclampsia, and the subtypes preterm and term, in a second pregnancy based on the presence of preeclampsia in the first pregnancy and the development of interpregnancy hypertension.

## MATERIALS AND METHODS

The Swedish National Board of Health and Welfare authorized access to a pseudo-anonymized database from the Swedish Medical Birth Register and the National Prescribed Drug Register (Dnr 218/251). In Sweden, antenatal care is standardized and free of charge. The Swedish Medical Birth Register gathers data for over 99% of all births in Sweden, including prospectively collected demographic data; information on reproductive and medical history; and complications during pregnancy, delivery, and the neonatal period.^22^ Information is documented by a midwife at the first antenatal visit and by the responsible doctor or midwife at discharge from the hospital after delivery. Copies of the standardized antenatal, obstetric, and neonatal records are sent to the Swedish Medical Birth Register. The National Prescribed Drug Register includes all dispensed prescribed drugs in Sweden, using the World Health Organization’s Anatomical Therapeutic Chemical Classification (ATC) codes. Statistics Sweden provided data from the Total Population Register and the Swedish Education Register. Linkage between the registers is possible due to the individual identification number given to all inhabitants in Sweden.

### Study population

**Figure 1.**
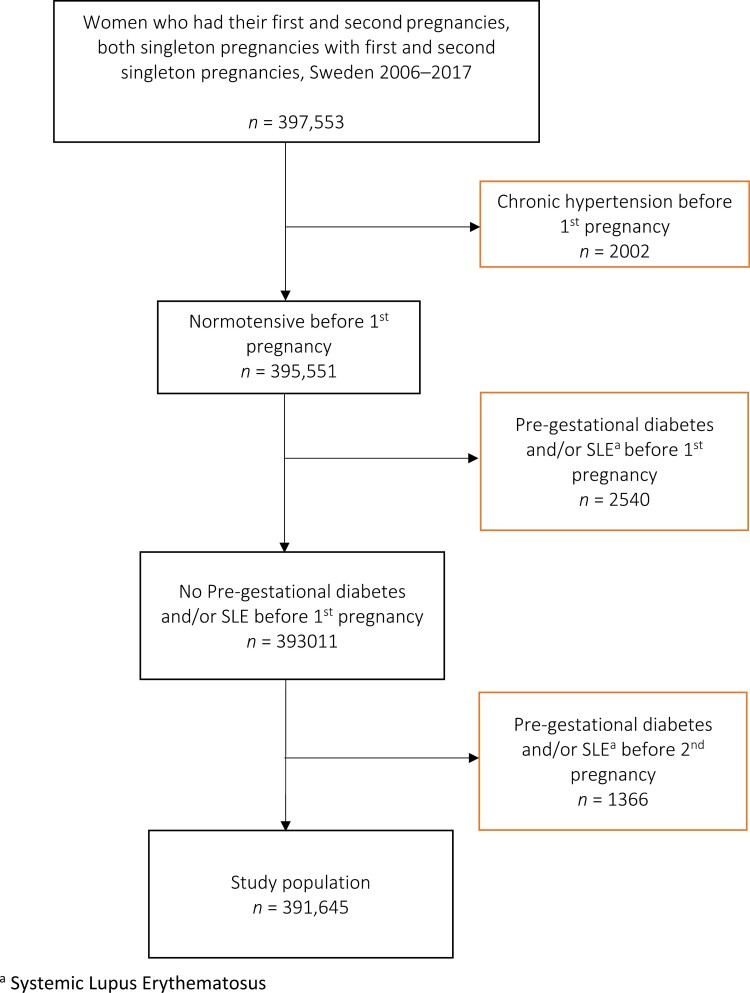
Study population.

The Swedish Medical Birth Register was used to define the study population. Data registered in the Swedish Medical Birth Register have a good degree of coverage, agreement, and internal validity compared to data from medical records.^23^ Approximately 1.5 million births at 22 weeks of gestation or later were recorded between 2006 and 2019. Of these, 397,553 were consecutive singleton births of a first and second child (Figure 1). Women with hypertension before their first pregnancy were excluded (*n* = 2,002). Information on chronic hypertension was collected at the first antenatal visit using checkboxes and identified at hospital discharge after delivery using the International Classification of Diseases (ICD)-10 codes (O10, O11, I10–15). The validity of self-reported hypertension is adequate, with a sensitivity of 81%, specificity of 95%, and positive predictive value of 87%.^24^ The sensitivity of the diagnosis of chronic hypertension at discharge from the hospital using ICD-codes is low in the general Swedish population.^25^ Although there is no study available on the validity of chronic hypertension during pregnancy in the Nordic countries, a recent study from the United States indicated a much higher validity in pregnancy with a sensitivity of 88% and a specificity of 98%.^26^ Both diabetes and systemic lupus erythematosus (SLE) are strong risk factors for preeclampsia, but preeclampsia does not predispose women to SLE or diabetes.^27,28^ To increase the homogeneity of the study population, we chose to exclude women with pre-gestational diabetes and SLE before either the first or second pregnancy (*n* = 3,906). They were identified at hospital discharge after delivery using ICD-10 codes (O240–243, E10–14, M32). The final study population included 391,645 women (Figure 1).

### Exposures

Exposures were preeclampsia in the first pregnancy and the development of interpregnancy hypertension. We categorized the exposures into three groups depending on whether they had one or both risk factors. Women with neither risk factor were included as a reference group. In Sweden, preeclampsia was defined as new-onset hypertension (systolic blood pressure ≥ 140 mm Hg and/or diastolic blood pressure ≥ 90 mm Hg at two subsequent measurements) combined with proteinuria (>0.3g/24 hours) after 20 gestational weeks between 2006 and 2018.^29^ During the same period, superimposed preeclampsia was defined as chronic hypertension with new-onset or increasing proteinuria after 20 weeks gestation. In 2019, the definition of preeclampsia was revised into new-onset hypertension after 20 gestational weeks in combination with end-organ damage, and superimposed preeclampsia was defined as chronic hypertension accompanied by at least one of the following: persistent hypertension despite at least two antihypertensive medications, new or increasing proteinuria or other sign of other end-organ damage. During the study period, the Swedish Society of Obstetrics and Gynecology (SFOG) recommended initiating treatment of hypertension during pregnancy when blood pressure was ≥150/100 mm Hg, aiming at blood pressure <150/80–100 mm Hg.^30^ Information on chronic hypertension was collected at the first antenatal visit using checkboxes and identified at hospital discharge after delivery using ICD-10 codes (O10, O11, I10–15). Preeclampsia was identified by ICD-10 codes (O11, O14, O15). There is no Swedish study on the validity of ICD-10 codes of preeclampsia, but a Finnish study showed medium sensitivity (80%) but high specificity (99%) for the preeclampsia diagnosis.^31^ The maternal care and birth registers in Finland and Sweden are very similar. Interpregnancy hypertension was defined as chronic hypertension during the second pregnancy and identified as described above.

### Outcomes

Our primary outcome was preeclampsia in the second pregnancy, identified by ICD-10 codes (O11, O14, O15). We categorized preeclampsia into preterm (delivery <37 gestational weeks) or term (delivery ≥37 gestational weeks) preeclampsia. Gestational age in Sweden is generally assessed by a first-trimester or early second-trimester ultrasound scan.

### Covariates

The covariates included were maternal age at first and second delivery, early pregnancy body mass index (BMI) in first and second pregnancy, country of birth, level of education, years of interpregnancy interval, change in BMI between pregnancies, and smoking status in first and second pregnancy. At the first antenatal visit, the woman was weighed and asked about height or measured, and BMI was calculated as weight in kilograms divided by the square of height in meters. Maternal country of birth and education level information was collected from the Total Population Register and the Swedish Education Register, respectively. The interpregnancy interval was measured in years between the first delivery and the estimated conception of the second child. Information on BMI change between pregnancies was calculated, and the women were divided into three groups depending on the distribution (±standard deviation [SD]), decreased; ≥ −2SD or more, increased; ≥ +2 SD and unchanged; > −2 SD and < +2 SD. Information on smoking status for the first and second pregnancy was collected at the first antenatal visit, and the women were divided into four groups depending on whether they smoked in either one, both, or none of the pregnancies. Further categorization of the covariates are presented in Table 1.

**Table 1.** Numbers and rates of preeclampsia at second pregnancy by maternal characteristics

Total (*n*)		Cases (*n*)	Rate (%)
Preeclampsia second pregnancy			
Preeclampsia 1st pregnancy			
Yes	15,183	2,106	13.9
No	376, 462	3,466	0.9
Aspirin prescription during 2nd pregnancy			
Yes	9,491	822	8.7
No	382,154	4,750	1.2
Age (years) 2nd pregnancy			
≤24	37,081	435	1.2
25–29	118,464	1,540	1.3
30–34	154,750	2,108	1.4
≥35	81,316	1,489	1.8
Early pregnancy body mass index (kg/m^2^) 2nd pregnancy			
<18.5	9,158	60	0.7
18.5–24.9	220,298	2,056	0.9
25–29.9	93,368	1,583	1.7
≥30	47,239	1,563	3.3
Missing	21,582	310	1.4
Country of birth			
Sweden	312,864	4,674	1.5
Other Nordic country	4,168	53	1.3
Other European country	11,388	121	1.1
Outside Europe	63,194	724	1.1
Education (years)			
<9	49,639	717	1.4
9–12	100,438	1,658	1.7
>12	239,796	3,173	1.3
Missing	1,772	24	1.4
Interpregnancy changes			
Chronic hypertension (developed since 1st pregnancy)			
Yes	1,552	182	11.7
No	390,093	5,390	1.4
Interpregnancy interval (years)			
≤1	70,122	733	1.0
1–3.9	282,259	3,968	1.4
4–6	33,047	727	2.2
≥7	6,128	144	2.3
Missing	89	0	0.0
Body mass index change			
Decreased ≥2 units	17,086	187	1.1
Unchanged	268,387	3,367	1.3
Increased ≥2 units	63,164	1,372	2.2
Missing	43,008	646	1.5
Total smoking status^a^			
1st pregnancy smoking—2nd pregnancy smoking			
No–No	343,638	4,829	1.4
No–Yes	4,282	53	1.2
Yes–No	8,299	132	1.6
Yes–Yes	10,046	110	1.1
Missing	25,380	448	1.9
Total	391,645	5,572	1.4

Abbreviation: *n*, number.

^a^At first antenatal visit.

Low-dose aspirin use (ATC code B01AC06) in the second pregnancy was defined as at least one dispensed prescription for aspirin registered in the Drug Register between 3 months before conception until childbirth. Low-dose aspirin is only available by prescription in Sweden.

### Statistics

We investigated the association between the two risk factors (previous preeclampsia and interpregnancy hypertension) on the outcome of preeclampsia in the second pregnancy by calculating relative risk (RR) with 95% confidence intervals (CIs) for the exposure groups, using women with neither risk factor as the reference group. The following confounders were identified using the directed acyclic graph model: maternal age in the second pregnancy, BMI in the second pregnancy, country of birth, education, interpregnancy interval, interpregnancy BMI change, and smoking status (Supplementary Figure 1). Adjusted relative risks (aRRs) with 95% CIs were calculated using generalized linear models.

Preeclampsia as an outcome was further divided into preterm and term disease.

During the study period, only women with strong risk factors for preeclampsia, for example, previous early onset (<34 gestational weeks) preeclampsia, were recommended low-dose aspirin for preeclampsia prevention in Sweden.^29^ Today’s guidelines are wider, and women with previous preeclampsia or with chronic hypertension are usually recommended aspirin prophylaxis.^32^ We performed a sensitivity analysis of women in the exposure groups who had received a low-dose aspirin prescription during the second pregnancy. Women without the two risk factors who did not receive a low-dose aspirin prescription during their second pregnancy were used as a reference group. We thereafter, re-ran the analyses.

We calculated Rothman’s excess risk due to interaction (RERI = RR_11_ − RR_10_ − RR_01_ + 1), attributable proportion (AP = RERI/RR_11_), synergy index (SI = (RR_11_ − 1)/(RR_01_ − 1) + (RR_10_ − 1)), and their CIs as described by Anderson et al. and Hosmer et al.^33–35^ These interaction indices were used to quantify the interaction between the two risk factors on an additive scale.

All analyses were performed using SPSS software version 27.

## RESULTS

The rates of preeclampsia in second pregnancy by maternal characteristics are presented in Table 1. Women with preeclampsia in their first pregnancy and women who developed interpregnancy hypertension had high rates of preeclampsia (13.9% and 11.7%, respectively). The rate of preeclampsia also increased with maternal age, BMI, longer interpregnancy interval, and increased BMI between pregnancies. Smoking had a slight negative effect on the preeclampsia rate.

Compared with women who did not have preeclampsia in their first pregnancy and did not develop interpregnancy hypertension, women with preeclampsia in their first pregnancy had an almost 13-fold increased risk of preeclampsia in their second pregnancy (0.9% vs. 13.6%; aRR: 12.83; 95% CI 12.09–13.62), and women who had developed interpregnancy hypertension had a 6-fold higher risk of preeclampsia in their second pregnancy (0.9% vs. 7.7%; aRR: 6.06; 95% CI 4.83–7.60, Table 2). Women with both risk factors had a 14-fold increased preeclampsia risk in a second pregnancy (0.9% vs. 21.5%, aRR: 14.51; 95% CI 11.77–17.89). The interactive indices suggested an antagonistic interaction on an additive scale, meaning that the combined effect of the two risk factors is less than the sum of their individual effects. [RERI = −3.38 (−6.71 to −0.05), AP = −0.23 (−0.51 to 0.04), SI = 0.76 (0.03–1.48)].

**Table 2.** Overall rate and risk of preeclampsia in second pregnancy by exposure group

Preeclampsia 1st pregnancy	Interpregnancy hypertension	Total (*n*)	Preeclampsia second pregnancy			
			Cases (*n*)	Rate (%)	RR (95% CI)	aRR^a^ (95% CI)
No	No	375,366	3,382	0.9	1.00 15.13 (14.36–15.95) 8.51 (6.91–10.48)	1.00
Yes	No	14,727	2,008	13.6		12.83 (12.09–13.62)
No	Yes	1,096	84	7.7		6.06 (4.83–7.60)
Yes	Yes	456	98	21.5	23.85 (19.95–28.52)	14.51 (11.77–17.89)

Abbreviations: *n*, number; RR, relative risk.

^a^Adjusted for maternal age second pregnancy, body mass index at first antenatal visit in second pregnancy, country of birth, education, interpregnancy interval, change in body mass index between pregnancies, and total smoking status at first antenatal visit in first and second pregnancies.

Compared with women who did not have preeclampsia in their first pregnancy and did not develop interpregnancy hypertension, women with preeclampsia in their first pregnancy and women who developed interpregnancy hypertension had a RR for preterm preeclampsia of 15.80 and 8.99, respectively, (Table 3). Women with both risk factors had a 5.5% recurrence rate and an aRR of 26.66; 95% CI 17.41–40.80 for preterm preeclampsia in a second pregnancy. For preterm preeclampsia, the interactive indices suggested a synergistic interaction on an additive scale between the risk factors [RERI = 2.87 (−9.32 to 15.06), AP = 0.11 (−0.33 to 0.55), SI = 1.13 (0.65–1.61)]. However, for term preeclampsia, the interaction between the risk factors seemed to be antagonistic on an additive scale [RERI = −3.91 (−7.52 to −0.30), AP = −0.29 (−0.72 to 0.11), and SI = 0.76 (0.48–1.04)].

**Table 3.** Rates and risks of preterm (<37 weeks) or term (≥37 weeks) preeclampsia by exposure group

Preeclampsia 1st pregnancy	Interpregnancy hypertension	Preeclampsia second pregnancy					
		Preterm			Term		
		Cases (*n*)	Rate (%)	aRR^a^ (95% CI)	Cases (*n*)	Rate (%)	aRR^a^ (95%CI)
No	No	626	0.2	1.00	2756	0.7	1.00
Yes	No	383	2.6	15.80 (13.71–18.21)	1625	11.0	12.95 (12.12–13.83)
No	Yes	24	2.2	8.99 (5.59–14.45)	60	5.5	5.61 (4.32–7.29)
Yes	Yes	25	5.5	26.66 (17.41–40.80)	73	16.0	13.65 (10.63–17.52)

Abbreviations: aRR, adjusted relative risk; CI, confidence interval; *n*, number.

^a^Adjusted for maternal age second pregnancy, body mass index at first antenatal visit in second pregnancy, country of birth, education, interpregnancy interval, change in body mass index between pregnancies, and total smoking status at first antenatal visit in first and second pregnancies.

In a sensitivity analysis of women in the exposure groups who were prescribed low-dose aspirin, the association between preeclampsia in both the first and second pregnancy was almost the same in women who developed interpregnancy hypertension and those who did not. Women without the two risk factors who did not receive a low-dose aspirin prescription during their second pregnancy were used as a reference group. The interactive indices between the two risk factors, preeclampsia in the first pregnancy and interpregnancy hypertension, suggested an antagonistic interaction concerning risk of preeclampsia in a second pregnancy [RERI = −9.97 (−16.73 to −3.21), AP = −0.67 (−1.34 to −0.02), and SI = 0.66 (0.26–1.06)] (Supplementary Table 1). We did not have sufficient power to calculate the interactive indices between the two risk factors for the outcomes of preterm and term preeclampsia in the sensitivity analysis.

## DISCUSSION

In this study, we demonstrate that the interaction between the two risk factors, preeclampsia in a first pregnancy and development of interpregnancy hypertension before a second pregnancy, was different depending on the preeclampsia subtype. For preterm preeclampsia, there were indications of a synergistic interaction between the two risk factors, amplifying the risk. In contrast, there was no difference in the risk of term preeclampsia in women with preeclampsia in first pregnancy who did or did not develop interpregnancy hypertension.

This study provides important information on the interaction between preeclampsia in a first pregnancy and the development of interpregnancy hypertension before a second pregnancy regarding the risk of overall, preterm, and term preeclampsia. The recurrence risk of preeclampsia has been studied extensively, and the rate in this study (14%) was in accordance with previous studies.^36–38^ Prior studies on the risk of preeclampsia in women with chronic hypertension have explored previous preeclampsia as a risk factor.^15,16,39^ Even though these studies are small in size, included women with varied parity, reflect previous preeclampsia not just in the most recent pregnancy, and varied in regard to the duration of chronic hypertension, the prevalence of recurrent preeclampsia in women with both risk factors in our study (21.5%) was in agreement with their findings.

The etiology of preeclampsia is complex and often divided into subtypes that have different associations with heritability, biochemical markers, clinical features, and maternal and infant outcomes.^40^ The most common classification of subtypes is based on gestational age at onset or at delivery. Preterm preeclampsia is strongly associated with defective placentation. In terms of preeclampsia, placentation is usually normal, but there are strong associations with maternal cardiometabolic variables that contribute to the pathogenesis later in the pregnancy.^41^ It is therefore tempting to assume that the risk of both preterm and term preeclampsia would be amplified in women with preeclampsia in their first pregnancy who develop chronic hypertension before their second pregnancy. But the findings of this study suggest that this is only true for preterm preeclampsia, where the two risk factors had a synergistic interaction.

The use of low-dose aspirin for preeclampsia prevention was less common in Sweden during the study period than it is today. It is likely that the women who did receive aspirin prophylaxis suffered from more severe chronic hypertension and/or preeclampsia in their first pregnancies.^32^

Similar to preterm preeclampsia, women with superimposed preeclampsia have higher complication rates during pregnancy and delivery, spend longer time in hospital, and are more likely to develop long-term cardiovascular sequelae after the pregnancy than preeclampsia patients who do not have pre-pregnancy hypertension.^42,43^ Bearing this in mind, our results suggest that the pathophysiology seen in superimposed preeclampsia might have more in common with preterm preeclampsia than generally assumed. In this study, the overall rate of superimposed preeclampsia in women with previous preeclampsia was 21.5%, whereas the rate of preterm preeclampsia in a second pregnancy was 5.5%. Effective blood pressure management in early pregnancy in women with chronic hypertension reduces the risk of superimposed preeclampsia.^44^

The registry-based design of this study constitutes both its strengths and limitations. Variables from the Swedish Medical Birth Register included in the study have been validated.^45^ Our use of a large nationwide cohort enabled us to study relatively rare exposures and subtypes of preeclampsia. In Sweden, maternal care is free of charge, therefore, selection bias is unlikely. All of the data, except that regarding chronic hypertension, were collected prospectively, thereby excluding recall bias. The diagnosis of chronic hypertension was an exception because it was partly collected as self-reported data by a midwife at the first antenatal visit. This could have led to both recall bias and misclassification bias, diluting the association between chronic hypertension and preeclampsia. Furthermore, information on the duration of chronic hypertension and its underlying etiology was not included in the study, although the duration was limited by the study design. We excluded women with pre-gestational diabetes and SLE to increase the homogeneity of the study population. The interactive indices (RERI, AP, and SI) assume that the interaction is additive and do not consider whether the interaction is multiplicative or more complex; furthermore, they do not provide information on underlying biological mechanisms. Using all three indices can lead to conflicting results, but we thought their use was appropriate to provide a comprehensive assessment of the interaction.

In conclusion, there is a high risk of recurrent preeclampsia, particularly preterm, in women with previous preeclampsia in combination with the development of interpregnancy hypertension. In order to minimize the disease burden and reduce the damage for each mother and their baby, these women must be identified early in pregnancy to enable preventive treatment and close surveillance during their pregnancies.

## Supplementary Material

hpae034_suppl_Supplementary_Figure_1

hpae034_suppl_Supplementary_Table_1
