# Supplementary figures and images for: Does Developing Interpregnancy Hypertension Affect the Recurrence Risk of Preeclampsia? A Population-Based Cohort Study
Source: Am J Hypertens. 2024 Mar 19;37(7):523–30. doi: 10.1093/ajh/hpae034 (PMC11176272; doi:10.1093/ajh/hpae034)

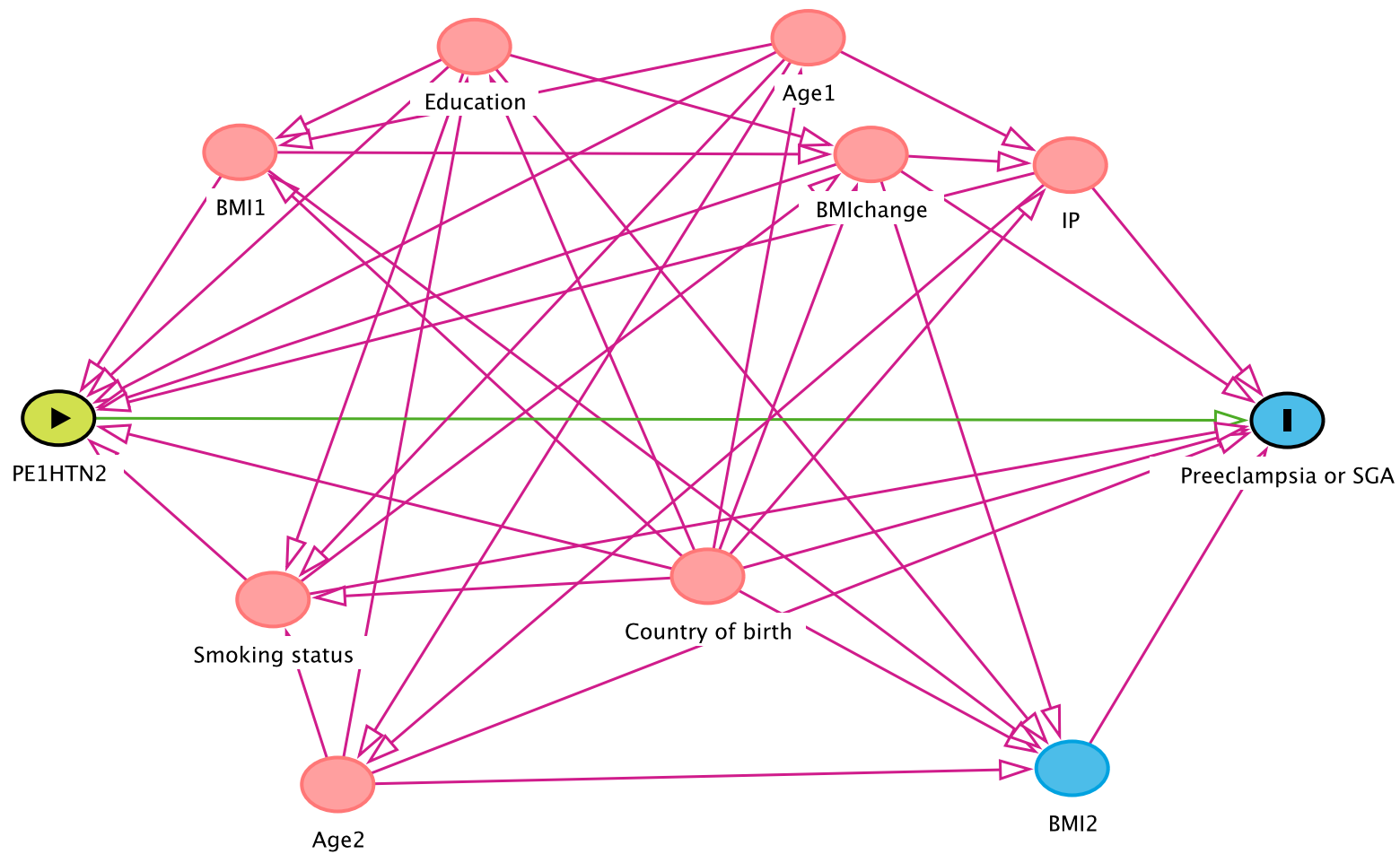

Supplement: hpae034_suppl_Supplementary_Figure_1 [file hpae034_suppl_supplementary_figure_1.pdf]
