# Supplementary material for: Does Developing Interpregnancy Hypertension Affect the Recurrence Risk of Preeclampsia? A Population-Based Cohort Study
Source: Am J Hypertens. 2024 Mar 19;37(7):523–30. doi: 10.1093/ajh/hpae034 (PMC11176272; doi:10.1093/ajh/hpae034)
Supplement: hpae034_suppl_Supplementary_Table_1 [file hpae034_suppl_supplementary_table_1.docx]

**Supplemental table 1**

Rate and risk of preeclampsia by exposure group in women who were prescribed low-dose aspirin

|  |  | |  | |  |  |  |  |  |
| --- | --- | --- | --- | --- | --- | --- | --- | --- | --- |
|  | |  | |  | |  | **Preeclampsia second pregnancy** | | |
| **Preeclampsia 1^st^ pregnancy** | | **Inter-pregnancy hypertension** | | **Aspirin 2^nd^ pregnancy^a^** | | **Total (*n*)** | **Cases (*n*)** | **Rate (%)** | **aRR ^b^ (95% CI)** |
| no | | no | | no | | 369,587 | 3229 | 0.9 | 1.00 |
| yes | | no | | yes | | 3429 | 612 | 17.8 | 16.56 (15.11–18.14) |
| no | | yes | | yes | | 119 | 14 | 11.8 | 9.11 (5.51–15.06) |
| yes | | yes | | yes | | 164 | 143 | 26.2 | 14.70 (10.51–20.57) |

n; number. CI; confidence interval

^a^ One or more prescriptions of aspirin in the time period between 3 months before conception until day of delivery.

^b^ aRR (Adjusted Relative Risk) Adjusted for maternal age second pregnancy, body mass index at first antenatal visit in second pregnancy, country of birth, education, inter-pregnancy interval, change in body mass index between pregnancies, and total smoking status at first antenatal visit in first and second pregnancies.
